# Supplementary figures and images for: Aging-related cerebral microvascular changes visualized using ultrasound localization microscopy in the living mouse
Source: Sci Rep. 2022 Jan 12;12:619. doi: 10.1038/s41598-021-04712-8 (PMC8755738; doi:10.1038/s41598-021-04712-8)

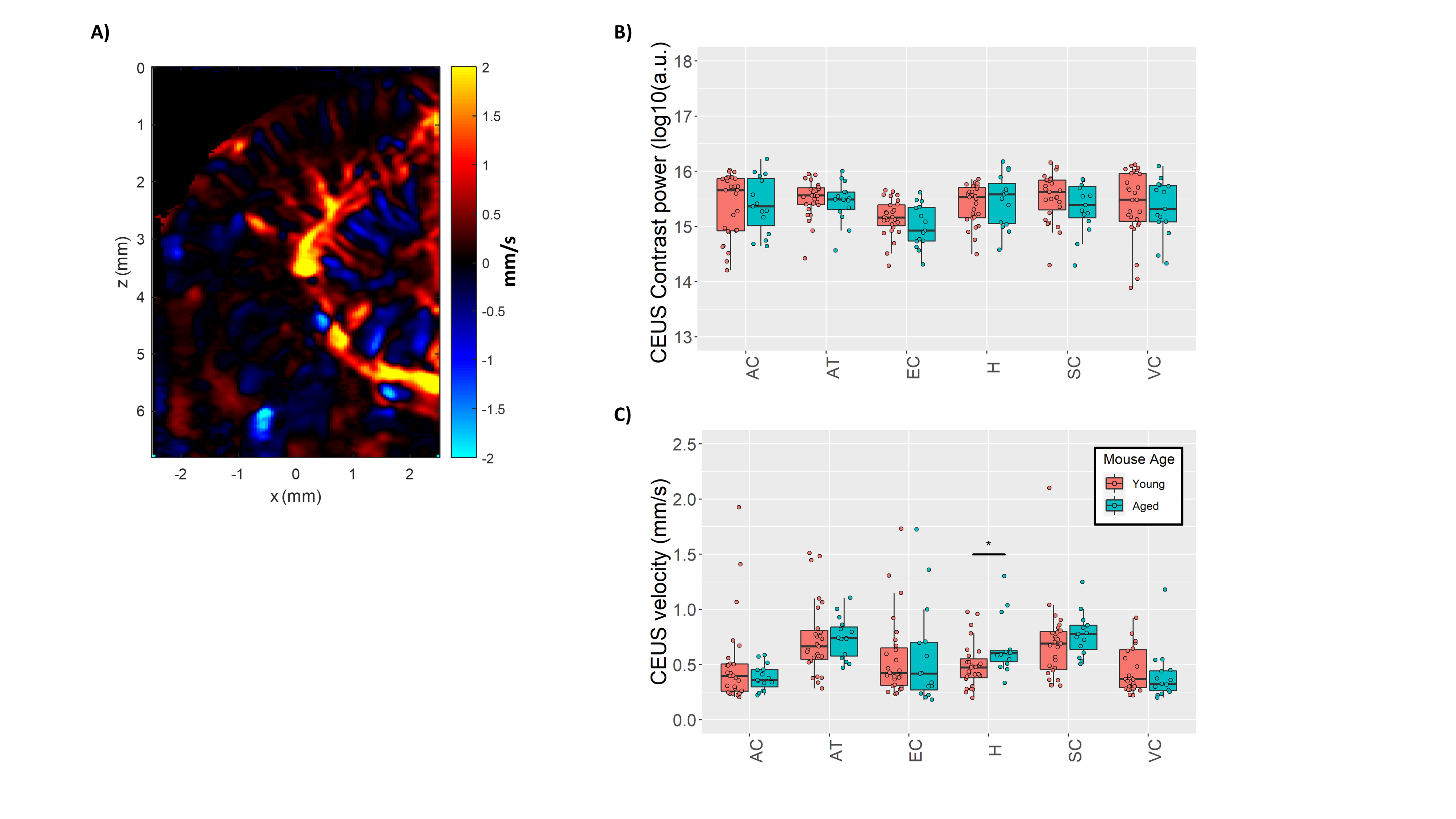

Supplement: Supplementary file 1 — Supplementary Information 1. [file 41598_2021_4712_MOESM1_ESM.png]

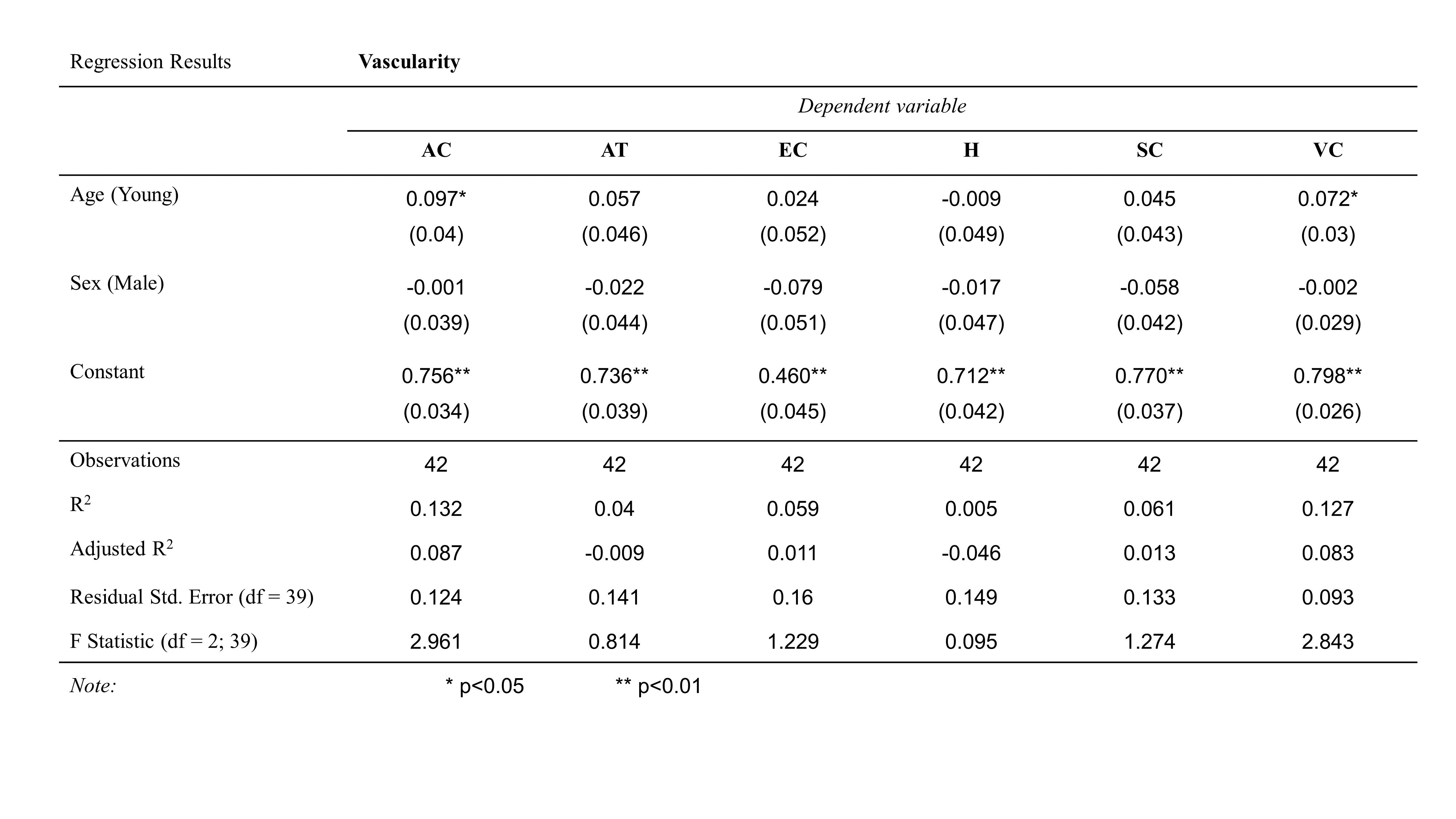

Supplement: Supplementary file 2 — Supplementary Information 2. [file 41598_2021_4712_MOESM2_ESM.png]

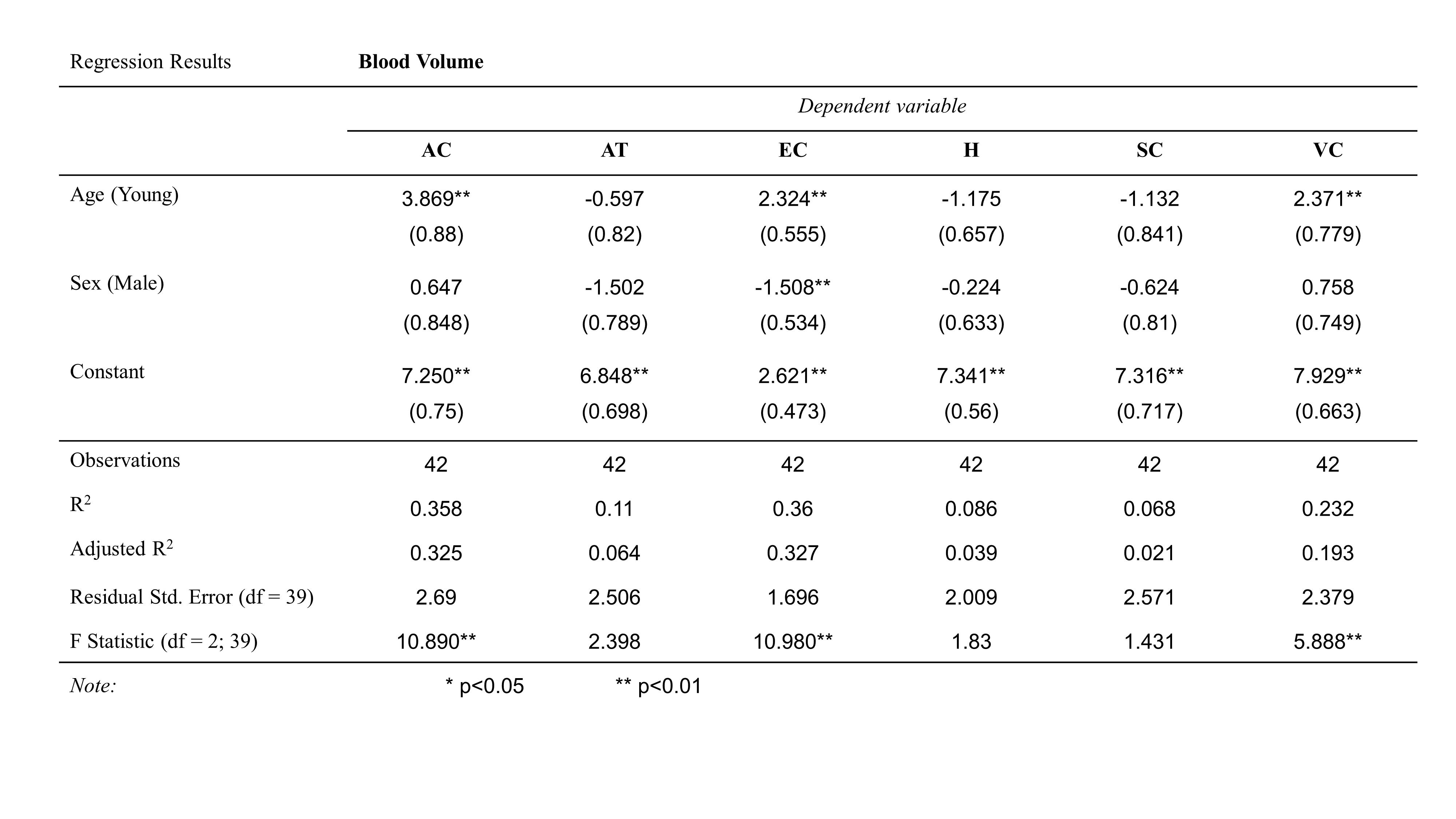

Supplement: Supplementary file 3 — Supplementary Information 3. [file 41598_2021_4712_MOESM3_ESM.png]

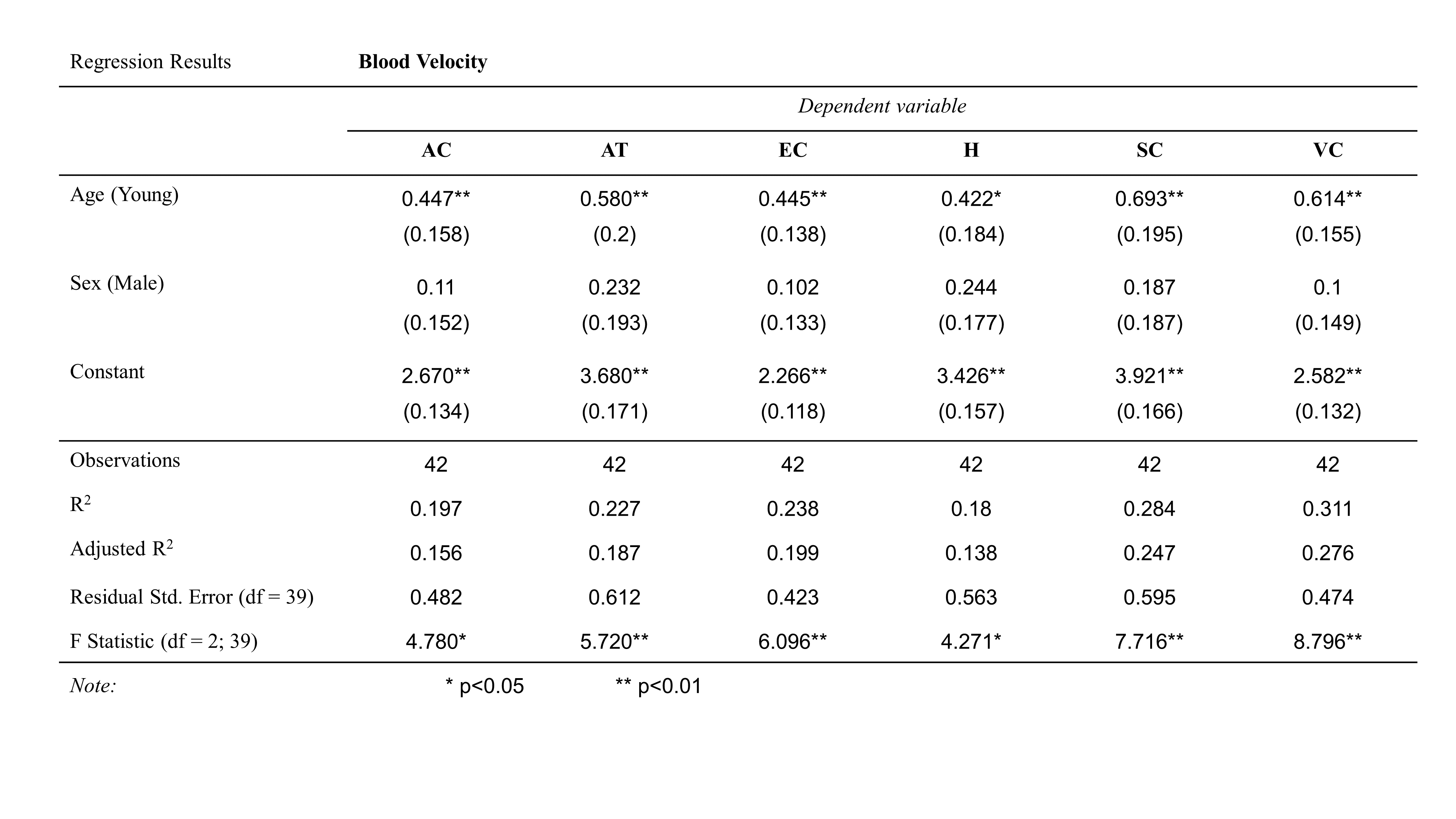

Supplement: Supplementary file 4 — Supplementary Information 4. [file 41598_2021_4712_MOESM4_ESM.png]

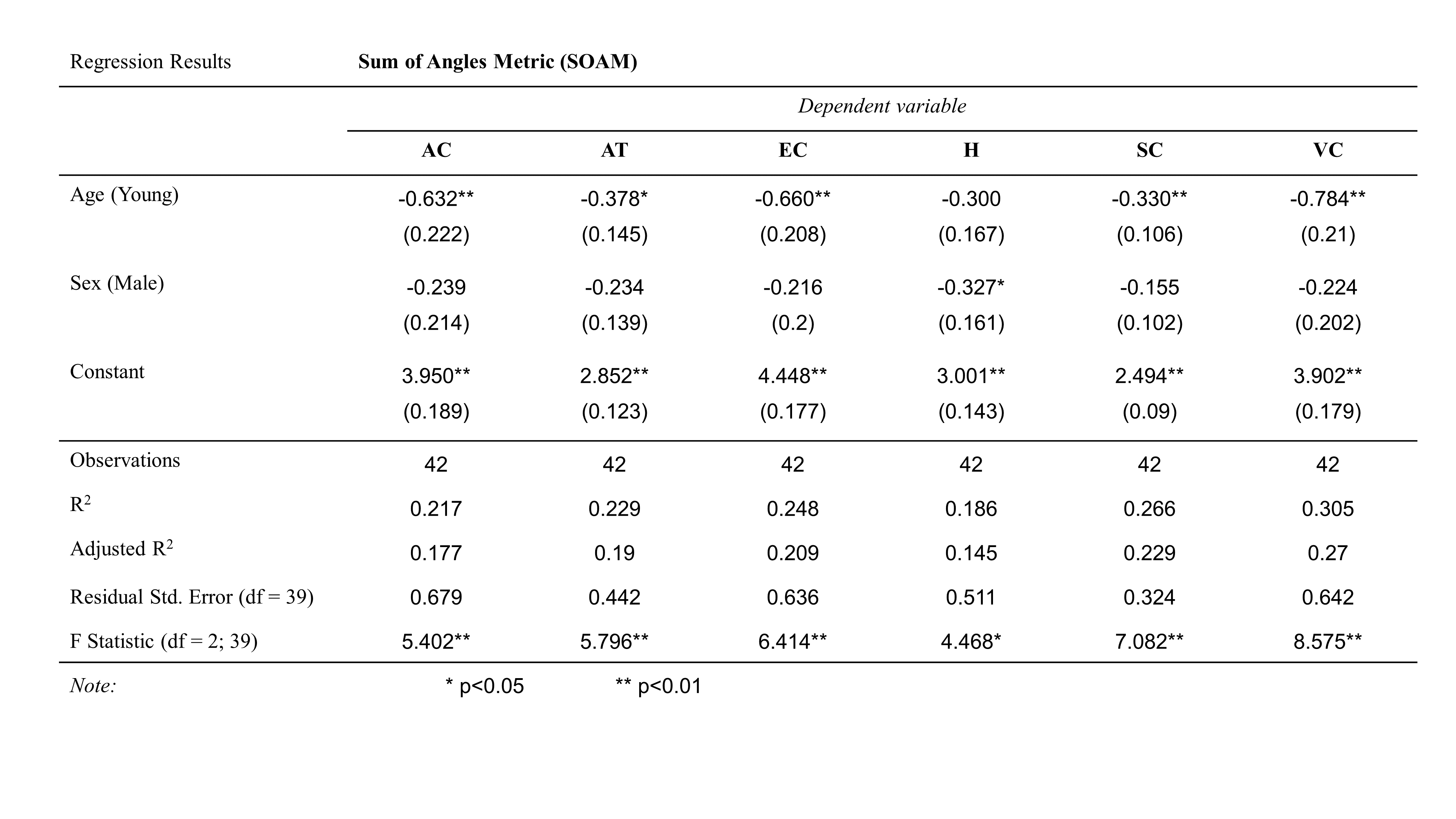

Supplement: Supplementary file 5 — Supplementary Information 5. [file 41598_2021_4712_MOESM5_ESM.png]
