# Supplementary material for: Aging-related cerebral microvascular changes visualized using ultrasound localization microscopy in the living mouse
Source: Sci Rep. 2022 Jan 12;12:619. doi: 10.1038/s41598-021-04712-8 (PMC8755738; doi:10.1038/s41598-021-04712-8)
Supplement: Supplementary file 6 — Supplementary Information 6. [file 41598_2021_4712_MOESM6_ESM.docx]

## Supplementary Figure 1 | Quantitative CEUS measurements

(**A**) Color-flow processing of mouse brain cross-sections demonstrated good visualization of the larger subcortical blood vessels, but limited ability to detect the slower flowing cortical vasculature. (**B**) Quantification of diffraction-limited contrast power images did not demonstrate a significant difference between the age groups. (**C**) Estimation of blood velocity demonstrated a significant increase (p = 0.026) in the hippocampus of the aged cohort. Color-flow image was rendered using MATLAB (R2019a, https://www.mathworks.com/).

## Supplementary Table 1 | Multivariate Analysis of Brain Vascularity

A multivariate linear regression with mouse age and sex as predictor variables for the ULM brain vascularity in each ROI.

## Supplementary Table 2 | Multivariate Analysis of Blood Volume

A multivariate linear regression with mouse age and sex as predictor variables for the ULM brain blood volume in each ROI.

## Supplementary Table 3 | Multivariate Analysis of Blood Velocity

A multivariate linear regression with mouse age and sex as predictor variables for the ULM brain blood velocity in each ROI.

## Supplementary Table 4 | Multivariate Analysis of Tortuosity

A multivariate linear regression with mouse age and sex as predictor variables for the ULM brain tortuosity, as measured by SOAM, in each ROI.
